# The Patient's Perspective on Shared Decision-Making in Advanced Parkinson's Disease: A Cross-Sectional Survey Study

**DOI:** 10.3389/fneur.2019.00896

**Published:** 2019-08-16

**Authors:** Frouke A. P. Nijhuis, Lieneke van den Heuvel, Bastiaan R. Bloem, Bart Post, Marjan J. Meinders

**Affiliations:** ^1^Department of Neurology, Canisius Wilhelmina Hospital, Nijmegen, Netherlands; ^2^Department of Neurology, Radboud University Medical Center, Donders Institute for Brain, Cognition and Behaviour, Nijmegen, Netherlands; ^3^Scientific Center for Quality of Healthcare (IQ Healthcare), Radboud University Medical Center, Radboud Institute for Health Sciences, Nijmegen, Netherlands

**Keywords:** advanced therapies, cross-sectional study, advanced parkinson's disease, shared decision-making, questionnaire

## Abstract

**Background:** Choosing between deep brain stimulation (DBS), Levodopa-Carbidopa intestinal gel (LCIG), or continuous subcutaneous Apomorphine infusion (CSAI) in advanced Parkinson's disease is a complex decision. It is paramount to combine evidence with the professional's expertise and the patient's preferences. The patient's preferences can be elicited and integrated into the treatment choice through shared decision-making (SDM).

**Objective:** In this cross-sectional survey study we explored patient's involvement in decision-making and identified facilitators and barriers for shared decision-making (SDM) in advanced Parkinson from the patient's perspective.

**Methods:** We invited 180 Dutch persons with Parkinson who started DBS, LCIG, or CSAI in the previous 3 years to complete a questionnaire. Questions covered three topics; (1) preferred and experienced roles in the decision process for an advanced treatment, (2) information needs to make a decision and actually received information, and (3) factors that had positively or negatively influenced shared decision-making (SDM).

**Results:** One hundred and twenty one participants completed the questionnaire. The large majority preferred to be involved in the decision-making (93%), and most respondents had experienced an active role (85%). In about half of the respondents (47%), their preferred role did not match their experienced role; 28% had a more active role than they would have preferred. Although 77% perceived to be fully informed at the time of decision, only 41% stated they knew all three therapeutic options. Participants identified the most important facilitators for shared decision-making (SDM) at the patient's level (i.e., perceiving the decision to be his own choice), at the neurologist's level (i.e., having expertise on all treatment options, and taking time for the decision), and within the professional-patient relationship (i.e., trust and having an open discussion). The main barriers for shared decision-making (SDM) existed at the patient's level (i.e., perceiving there is no choice), neurologist's level (own treatment preference), and organizational level (i.e., no research available that compares treatments, multiple professionals involved, and lack of consultation time).

**Conclusions:** Patients want to be involved and feel involved when choosing an advanced treatment, but often do not know all treatment options. Implementation of true patient involvement needs personalized information provision on all treatment options and improvement on how this information is communicated.

## Introduction

Parkinson's disease (PD) is a highly complex, multidimensional disease. With disease progression, severe motor and non-motor symptoms can develop with unpredictable on- and off fluctuations and dyskinesia. When these motor complications arise, advanced treatment options, including deep brain stimulation (DBS), Levodopa-Carbidopa intestinal gel (LCIG), and continuous subcutaneous Apomorphine infusion (CSAI) are important to consider. Each of them has specific advantages and disadvantages. Choosing a therapy, therefore, requires a careful deliberation process, in which the available scientific evidence, the clinician's expertise and the individual patient's characteristics and preferences are balanced and jointly guide the decision (1).

The application of these evidence-based medicine principles to this particular decision appears to be challenging in clinical practice. First of all, unbiased and comparable information for all three options is not straightforward, as no randomized controlled trials have been conducted including all three advanced treatments (2). Secondly, the clinician's expertise is often limited due to a lack of treatment experience or availability of the advanced treatments at their hospital (3). Third, including the patient's individual preferences is recognized as an ethical imperative, but it's questionable to what extent patients are actually actively involved (4, 5). To overcome the last challenge, shared decision-making (SDM) can play a pivotal role (6).

SDM means that the neurologist and the patient identify the decision to be made, share the treatment options and available evidence, elicit the patient's preferences and reach a shared decision (6–8). PD patients in general have indicated that they want to be more involved in clinical decision-making, but lack sufficient information to do so (9). In contrast, professionals often report that patients do not want to be involved in decision-making, which seems to be more common in older and less educated patients (10). More specifically, neurologists mention limited decision-making capacity in PD patients as an additional barrier to SDM in advanced PD, related to disease severity, and associated cognitive deficits (5). Quantitative studies investigating the patient's perspective on involvement in decision-making in this specific group of advanced PD patients have not been conducted.

The objective of this study was to investigate the patient's perspective on; (1) the preferred and experienced level of involvement in the decision process for an advanced treatment, (2) the information needs and received information for this decision, and (3) perceived facilitators and barriers for shared decision-making for this specific decision.

## Materials and Methods

### Questionnaire

The aim of the questionnaire was to systematically collect experiences of the decision-making process for an advanced treatment option. Previously conducted focus groups and interviews with advanced PD patients treated with an advanced therapy (CSAI, DBS, or LCIG) defined the content of the questionnaire (5). Items covered socio-demographics plus disease-related characteristics and three key topics; (1) role preferences and experienced roles in the decision process, (2) information needs to make a decision and actually received information, and (3) factors that had positively or negatively influenced shared decision-making (Supplement 1).

We recorded socio-demographics and disease-related information: years of PD disease duration, year of starting advanced treatment (in case of multiple advanced treatments, year of starting last treatment), type of advanced treatment received (multiple options possible), and disease severity (self-reported Hoehn and Yahr score) (11).

To obtain information about the decision-making process, we included the validated Control Preferences Scale (CPS), which captured the role preference, and experienced role in decision-making (12). This single item five-point scale consists of pictures combined with descriptions displaying five roles of the patient in decision-making (fully active, semi-active, collaborative, semi-passive, fully passive). The participant was asked which role (s) he would have wanted (preferred role) when the advanced therapy was chosen and which role (s) he actually had (experienced role). To evaluate the information received and the information needs, we asked which options were known at the time of the decision and how well-informed they felt on 22 information topics at the time of the decision (5). To determine if information needs and received information matched, we used the Importance Performance Analysis (IPA) (13, 14). Patients first rated the importance of each information attribute (the importance score) on a five-point Likert scale (not important, of little importance, neutral, important, very important). Next, they were asked whether they received sufficient, insufficient or no information on each attribute (the performance score). Finally, 33 items about facilitators (*n* = 19) and barriers (*n* = 14) for SDM, derived from our earlier study (5), were presented. For each item we asked to what extent it had been a facilitator/barrier for shared decision-making, on a five-point Likert scale (no opinion, no facilitator/barrier, small facilitator/barrier, moderate facilitator/barrier, large facilitator/barrier). The facilitators and barriers were divided into four categories (adapted from Grol and Wensing) (15); professional's level, patient's level, the professional-patient relationship level (social context level), and the organizational level. The professional level represented both the neurologist and PD nurse specialist, unless it was specifically stated neurologist. Patients had the possibility to add facilitators and barriers that were not included in the list. We designed two versions of the questionnaire, in which the order of the questions were different to prevent framing due to question order (16).

The questionnaire was piloted in eight persons; four patients, one expert in health education & communication, two lay people and an industrial designer, specialized in consumer process evaluation. Piloting addressed readability, comprehensibility and time to complete the questionnaire. Based on their feedback, the questionnaire was finalized.

### Target Population and Recruitment

Eligibility criteria for patients to participate were: (1) diagnosis of PD by a neurologist and (2) having started one of the advanced treatments (CSAI, DBS, or LCIG) in the past 3 years. We recruited participants via 13 neurologists, working in community (*n* = 4), regional (*n* = 4) and academic (*n* = 5) hospitals. We used purposive sampling to select neurologists with expertise on one or more advanced treatments who could include eligible patients. Neurologists were asked to send a paper-based questionnaire with a hyperlink to the online questionnaire to all their patients who had started an advanced therapy in the previous 3 years. To reduce a potential selection bias by the neurologists, the Dutch Parkinson's Disease Association published a recruitment advertisement on their website. Eligible patients could apply and subsequently received the paper-based questionnaire and a hyperlink to the online questionnaire. No reminders could be sent, as questionnaires were processed anonymously.

### Ethical Considerations

The regional medical ethical commission approved the study (CMO Nijmegen-Arnhem, registration number 2011-085). We asked all participants to give written informed consent prior to completing the questionnaire.

### Data Analysis

Data concerning the socio-demographics, disease characteristics and the facilitators/barriers were analyzed descriptively with frequencies and percentages. To investigate the congruence between preferred and experienced role in the decision-making process, we divided the number of participants whose preferred and experienced role exactly matched by the total number of participants. The mismatch and direction of mismatch was calculated by counting the participants whose preferred role differed from the experienced role in decision-making (either preferred a more active or a more passive role than experienced) divided by the total number of participants. We analyzed group differences in gender, age, education, and disease severity between the group with a matched role vs. the group with a mismatch, using Pearson's χ-square test or Fisher exact test. We calculated the correlations between the patients' knowledge of existing treatment options (not knowing all options vs. knowing all options) and whether they felt fully informed (yes or no) at the time of decision using Pearson's χ-square test.

For the IPA analysis, the performance rates were recalculated into a five-point Likert scale, allowing the comparison of the importance scores with the performance scores for the IPA. The mean importance score of all information attributes was calculated, as well as the mean performance score of all attributes. Subsequently, the mean importance score and mean performance score for each information attribute separately was calculated. To prioritize the attributes that need improvement, the attributes were ranked on importance and performance using the mean of each attribute on importance and compare it to the mean importance score of all attributes together. Attributes that scored higher than the mean importance of all attributes, and had a mean performance score which was lower than the mean importance score, require improvement (13, 14).

For all analyses, we used SPSS, version 25. Because of large numbers of missing data for the facilitators and barriers, missing value analysis was conducted using Pearson's χ-square test or Fisher exact test for analyzing differences between completers and non-completers, without applying imputation methods.

## Results

A total of 121 patients completed the questionnaire. Most of them (*n* = 112) replied to the invitation from the neurologists (171 invitations sent out; response rate 65%). Of these 121 completed questionnaires, we excluded 10 (= 8%) as they either did not fill in the date when they had started the treatment (*n* = 6) or the treatment started more than 3 years ago (*n* = 4). The remaining 111 questionnaires were included for analysis. Forty percent of the participants had filled in the questionnaire alone, 46% with their partner, and 13% with someone else (mostly a family member). One participant had not filled in this question. See Table 1 for demographics and disease characteristics.

**Table 1:** Demographics and disease characteristics.

	**Sample size**	**Value**
Gender [men, *n* (%)]	111	68 (61)
Age in years [median (range)]	111	65 (38–84)
Work [*n* (%)]	110	
Not working		88 (80)
Part-time job		17 (15)
Full-time job		5 (5)
Marital status [*n* (%)]	110	
Single		22 (20)
Relation/married, living together		84 (76)
Relation, living apart		4 (4)
Education [*n* (%)]	109	
Primary school		10 (9)
Secondary school		41 (38)
Lower vocational education		30 (27)
Higher education		28 (26)
PD disease duration [*n* (%)]	111	
5–10 years		31 (28)
More than 10 years		80 (72)
Self-reported Hoehn and Yahr stage [*n* (%)]	109	
0		3 (3)
1		13 (12)
2		9 (8)
3		52 (48)
4		24 (22)
5		8 (7)
Treatment [*n* (%)^*^]	111	
CSAI		20 (18)
DBS		65 (59)
LCIG		42 (38)
Years of treatment	111	
0–1		19
1–2		60
2–3		32

*CSAI, continuous subcutaneous Apomorphine infusion; DBS, deep brain stimulation; LCIG, Levodopa-Carbidopa intestinal gel*.

* *The total number exceeds 111 as some patients had undergone more than one of the advanced treatment options*.

### Roles in Decision-Making

In retrospect, the vast majority preferred to have been actively involved in the decision-making process; 31% of the participants wanted to decide themselves and 62% wanted it to be a shared-decision together with their neurologist. Many patients also had experienced an active role; 41% perceived they had decided themselves and 43% experienced shared decision-making. Half of the respondents (47%) experienced a mismatch between their preferred and experienced role in decision-making (Table 2). In those cases where there was a mismatch, 28% of the respondents experienced a more active role in the decision-making process than preferred, and 19% experienced a less active role than preferred. Of those who would have preferred a less active role, the majority (62%) had a preference for shared decision-making but experienced that they had made the decision themselves. Among the patients that would have preferred a more active role, 65% preferred shared decision making but experienced that the neurologist took the decision for them. The patients who experienced a match between their preferred and experienced role in the decision-making process did not differ from patients who experienced a mismatch, in terms of gender, age, education, or disease severity.

**Table 2:** Congruency between preferred and experienced roles in decision-making.

		**Experienced role**					
		**Patient alone**	**Patient with neurologist input**	**Shared decision**	**Neurologist with patient input**	**Neurologist alone**	**Total number of patients (%)**
**Preferred role**	Patient alone	**2**	–	–	–	–	2 (2)
	Patient with neurologist input	7	**12**	6	–	2	27 (29)
	Shared decision	4	13	**31**	8	1	57 (62)
	Neurologist with patient input	–	–	2	**3**	–	5 (6)
	Neurologist alone	–	–	–	–	**1**	1 (1)
	Total number of patients (%)	13 (14)	25 (27)	39 (43)	11 (12)	4 (4)	92 (100)^*^

*Preferred role is: the role a patient would have wanted in the decision-making process; perceived role is the role the patient has experienced in the decision-making process. Blue cells are the cells that represent matched roles*.

* *13 patients did not fill in a perceived role and six patients had invalid data for one or both questions on the roles in decision-making*.

### Knowledge, Information Needs, and Information Support

Although the majority of patients (77%) felt fully informed at the time of the decision, only 41% was aware of all three treatment options. Overall, DBS was the best-known treatment, with 83% knowing this option before the decision was made. LCIG was second with 69% and 58% of the patients knew CSAI was an option. CSAI patients were the least informed about the alternative options (Table 3). In each treatment group, a few patients (CSAI and DBS one patient; LCIG three patients) indicated that they did not know the treatment they eventually started. Knowing all options was not associated with feeling fully informed [$X_{\left(1\right)}^{2}$ = 0.001, *p* = 0.590].

**Table 3:** Degree of known advanced treatment options.

	**Known treatments before decision**			
**Chosen treatment**	**CSAI**	**DBS**	**LCIG**	**Total**
CSAI	7 (88)	3 (38)	3 (38)	8 (9)
DBS	29 (53)	54 (98)	32 (58)	55 (58)
LCIG	14 (45)	23 (74)	28 (90)	31 (33)
Total	50 (58)	80 (83)	63 (69)	94 (100)

*Patients could have knowledge of more than one treatment, therefore percentages of known treatments are more than 100 percent. CSAI, continuous subcutaneous Apomorphine infusion; DBS, deep brain stimulation; LCIG, Levodopa/Carbidopa intestinal gel*.

**Figure 1:**
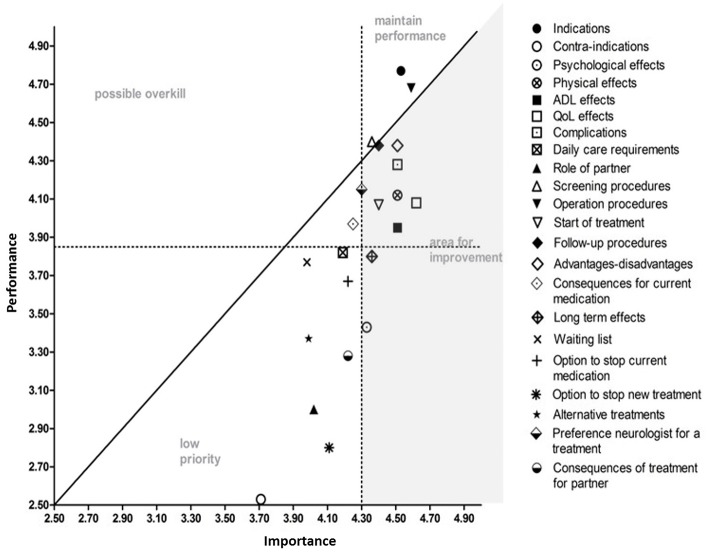
Importance and Performance (IPA) analysis. All information attributes are displayed in random order. The attributes in the gray area represent the information attributes that need improvement. The attributes in the area of “maintain performance” scored high in importance and information provision; those attributes are sufficiently covered. The attributes in the area of possible overkill' are attributes that were considered less important but were covered adequately in information provision. The area of “low priority” represents the attributes that were not sufficiently covered in information provision but were also considered less important. The information attributes are described in more detail in the questionnaire (Supplement 1).

When asked to indicate how important they valued the information attributes (importance) and whether or not they had received enough information on these attributes to make a decision (performance), all attributes were found to be important, except for information on contra-indications (<50% of respondents). Information about effects of the advanced treatments on quality of life was found to be most important. The IPA analysis identified nine out of the 22 information attributes that were considered to be important, while information provision had been inadequate (Figure 1). The lowest performance scores were given for information on long term effects and psychological effects of the advanced treatments.

### Facilitators and Barriers to Shared Decision-Making

Among the five most important facilitators for SDM, two related to the professional: (1) the experience of the neurologist with the treatments (89% of patients considered this to be a moderate-to-large facilitator), and (2) the neurologist taking time for the decision (83%). Two factors related to the professional and patient interaction: (1) trust between the patient and professional (84%), and (2) an open discussion between the patient and professional (80%). One facilitator related to the patient: the patient considering the decision his own choice (83%).

Participants ranked barriers frequently as no barrier (on average 54% per barrier, range 43–66%) or had no opinion (on average 22%, range 12–35%). The five most important barriers were more diverse as they related to the patient, the professional, and the organizational level. Patients identified the perception of having no choice as the most important barrier (24% of respondents considered this a moderate to large barrier). The professional having his own preference for a treatment (23%) was the barrier at the professional level. Furthermore, three factors at the organizational level emerged: (1) lack of time at the consultation to discuss the options (21%), (2) not having the same professional involved during the decision process (19%), and (3) the lack of research to compare the treatments (22%).

On average, barriers had 12% missing values (range 10–14%) and for the facilitators this was 13% (range 10–16%). The missing value analysis showed that two patients had not ranked any of the facilitators or barriers. Ten respondents had more than 50% percent missing values for both the barriers and facilitators. This group of 10 respondents did not differ from the other 101 respondents in terms of age, educational level, or disease severity. There were relatively more women in the group with more than 50% missing values compared to the other group (*p* = 0.44, Fisher's exact test).

## Discussion

Persons with PD want to be actively involved in the decision-making process for an advanced treatment. This observation is in line with the notion that persons with PD in general want to be involved in decision-making (9, 17). However, it contrasts with a study showing that persons with PD prefer a less active role when confronted with hypothetical decisions, when their illness progresses (18). The latter applies to our population of people with advanced PD, of whom 72% had a disease duration of more than 10 years. One of the reasons for a less active role when disease progresses is that patients develop cognitive impairments which would limit their decision-making capacities (5, 18, 19). It is possible that, due to selection bias in our population, our respondents did not have cognitive deficits. No reliable test for subjective cognitive impairment in PD is available yet and objective cognitive tests are impossible in a self-reported questionnaire (20). However, mild dementia is considered to be one of the most important non-motor symptom indicators of advanced PD (20, 21), and it is therefore likely that participants in our study will have at least some degree of cognitive impairment.

A striking observation is that, although participants experienced active involvement, only half of them experienced the role they preferred. In those cases where the preferred and perceived role did not match, most patients experienced a more active role than preferred. In more detail, most of these patients had preferred shared decision-making. Instead, they experienced taking the decision themselves, with or without input from the neurologist. It could be a reflection of the experience that patients did not feel supported by the professional when making the decision. It highlights the importance of not abandoning patients to take the decision alone. Instead, one should strive for shared decision-making, where patients are supported in exploring their needs and contributing their expertise to the conversation (22). It could also mean that we should not force an active role for all patients, as this could cause distress in patients who genuinely want a less active role (8). We did not ask the patients explicitly if they wanted the partner/ caregiver to be involved, because we evaluated that in our qualitative analysis. We did see that partners and other caregivers are highly involved as the majority of participants filled in the questionnaire with a caregiver. The role of the partner is known to be increasingly important for patients in the advanced stage and lack of support from a caregiver could be a contraindication for an advanced therapy (23). Furthermore, caregivers also participate more actively in treatment decisions when cognitive impairments increase in the advanced stage (24). This implies that SDM should be individualized and patients, caregivers and neurologists should explicitly discuss which role the patient prefers in the decision process.

To make a shared decision, patients need to know all available options. Even though patients felt fully informed, only 41% knew all options. Patients have indicated before that they were not informed about the advanced treatment options by their physician (25) and they preferred to receive more information on the treatment (26). This raises the question whether patients can truly be involved when they do not know all options.

A more striking finding is that the patient is seemingly unaware of the missing information as they feel fully informed and, at the same time, they do not know all options, which could result in the so-called “silent misdiagnosis” (27). This phenomenon describes the misdiagnosis about the treatment preference. Similar to the medical misdiagnosis, the preference misdiagnosis also harms the patient, when the chosen treatment does not fit that individual patient. Once patients are better informed, they often choose a different therapy (27). PD patients in the advanced stage without advanced therapy indicated that the reason they were not on advanced therapy was that they needed more time to decide (23). It is not clear why the patients needed more time to decide, but it suggests a need for better guidance during the decision-making process.

When we evaluated which information is needed to make the decision, PD patients showed very diverse information needs and they considered almost all attributes to some extent important. Patients weigh the benefits and harms of the options differently (17), which means there is no general information strategy that can be applied. This requires an individual approach to identify the key information attributes that need to be discussed with each patient.

The information on effects of the advanced treatments on quality of life had the highest importance score and scored low on performance. The lowest performance scores were given for information on long term effects and psychological effects of the advanced treatments. Clinicians can use these outcomes on performance scores as a starting point to improve the information provision and can use their skills to adjust the information to the needs to the individual patient.

Improving the knowledge of patients on all options alone, however, will not be sufficient to improve SDM, as other factors also facilitate or hinder SDM according to the participants. The facilitators in the top five were at the patient's level, professional's level, and the level of the patient- professional interaction. Patients noted themselves that if they felt that it is their decision, this supports them to actively participate in decision-making. It requires a context where the patient feels empowered to uptake that active role (28). At the professional level, the experience of the neurologist with the treatments was considered a very important facilitator and the professional taking time for the decision process also stimulated the patient to become involved. The facilitators at the interpersonal level include trust between the patient and neurologist, and having an open discussion in the decision process. Because PD patients who are eligible for an advanced treatment often already have a well-established and trustful relationship with their neurologist, this is a good foundation for SDM (26).

Unlike the agreement among patients regarding factors that facilitate SDM, they did not often point out factors that hampered SDM. One of the reasons could be that a large percentage of our population experienced an active role and therefore did not experience barriers to SDM. The top five barriers represented the patient's level (i.e., patient feeling there is no choice), professional's level (i.e., the professional's own treatment preference), and at the organizational level (i.e., lack of time during consultation, multiple professionals involved, and lack of research on all treatments). The most frequently rated barrier by the patients was that they did not experience a choice. The observed lack of knowledge about all treatment options among patients might have induced the feeling that there was nothing to choose from. Various factors were identified that contribute to this knowledge gap: first of all, there is no evidence from studies that directly compare the three treatments. Secondly, the neurologist can have limited experience with one or two treatment options, for example because not all options can be offered in their own hospital (5). Thirdly, when the neurologist has a clear own preference, the information sharing will inevitably be biased. All these factors increase the power imbalance between the patient and professional, hampering SDM (28, 29).

To improve the implementation of SDM for choosing an advanced treatment, steps have already been taken to improve the neurologist's expertise by defining patient profiles for suitability of the advanced treatments (21). The information for the patient should also be provided in a transparent way, to reduce the influence of the (possible biased) experience of the individual neurologist with the treatments. There is a need for more guidance during this decision process to prevent patients from deferring a decision or not make a decision for an advanced treatment at all (23). This can be realized by developing a decision support tool, for example, that presents the options in a clear, meaningful manner, and supports patients in constructing their preferences (30). Additionally, the interactional context between patient and professional is equally important to improve SDM. From our facilitator ranking exercise, it appeared that training professionals to improve their interpersonal skills is needed. To improve the communication during the decision-making process, the neurologist and PD nurse specialist should be trained in skills to support and facilitate the patient to express their needs and preferences and to clarify which role the patient prefers. Improvements in the organizational structure are necessary to give time for the decision process and to have a limited number of professionals involved. However, what type of trainings or interventions are most effective to increase the use of SDM by neurologists is unclear due to low level evidence (31) and should be further investigated.

### Strengths and Limitations

The major strength of this study is that it quantitatively analyzed the decision-making process for advanced treatments in PD. Earlier, we qualitatively evaluated the decision-making process, which led to a large set of information needs that could be improved, as well as a large number of facilitators and barriers to improve the implementation SDM. In the current study, we prioritized information needs and barriers and facilitators for SDM, which helps us to develop a tailored implementation strategy.

A limitation of the study is that we asked the preferred and experienced roles in decision-making retrospectively. To increase the number of available patients, we included patients that started treatment in the previous 3 years. This, however, increases the risk of recall bias. For the congruence between the preferred role and the perceived role, specifically, we found that in other retrospective studies this congruence was slightly higher with 63% (32). It is argued that congruence increases in retrospective studies because of the decision evaluation of the patient and the desire of the patient for congruence, introducing cognitive bias (32). This could mean that the congruence in our population is an overestimation, compared to if we would have tested it prospectively.

Another limitation is the lack of knowledge on the cognitive status of our participants. As moderate to severe dementia is considered a contraindication for all therapies (21), we can assume that the patients did not have severe dementia at the time of decision. However, we cannot exclude that some patients have developed dementia after start of treatment and that this would have influenced their responses. In our ongoing prospective observational study of the decision process for an advanced treatment, we therefore evaluate the cognitive status of all participants (33).

Finally, many respondents (up to 16%) did not rate the list of facilitators and/or barriers for SDM. We could not find different characteristics for the group with most missing values compared to the rest of the participants, except for gender. An explanation for the large number of missing values for this topic in the questionnaire could be response fatigue, as the rating of the barriers and facilitators was one of the last topics (34). We anticipated partly on this beforehand with changing the order of barriers and facilitators in the two versions of the questionnaire. Another explanation could be that the questions were unclear or that the concepts of barriers and facilitators to SDM were difficult to understand, however, we clarified this concept in the questionnaire and no difficulties in understanding the questions emerged in the pre-test. As the missing values were equally divided across all items, they do not affect the interpretation of the importance ranking of each facilitator/barrier.

## Conclusions

Our study has shown that patients highly value SDM when choosing an advanced treatment option. The study also has revealed the current limitations of patient participation. One of the most important risks is that patients feel involved but are unaware of all options and information needs are not adequately addressed. Improvement of patient involvement could be achieved by creating a decision support tool that provides balanced information on all options, increasing the expertise of the neurologist, increasing the SDM skills of the neurologist, and improving the organizational context. How this can be best achieved has to be evaluated in further research.

## Data Availability

The dataset for this study is available upon request from the first author.

## Author Contributions

FN, BB, BP, and MM conceived the study. FN collected the data. FN and LvdH performed the statistical analyses. All authors reviewed and revised the manuscript for intellectual content.

### Conflict of Interest Statement

The authors declare that the research was conducted in the absence of any commercial or financial relationships that could be construed as a potential conflict of interest.
