# Supplementary material for: The Patient's Perspective on Shared Decision-Making in Advanced Parkinson's Disease: A Cross-Sectional Survey Study
Source: Front Neurol. 2019 Aug 16;10:896. doi: 10.3389/fneur.2019.00896 (PMC6706819; doi:10.3389/fneur.2019.00896)
Supplement: Supplementary file 1 [file Table_1.DOC]

RR
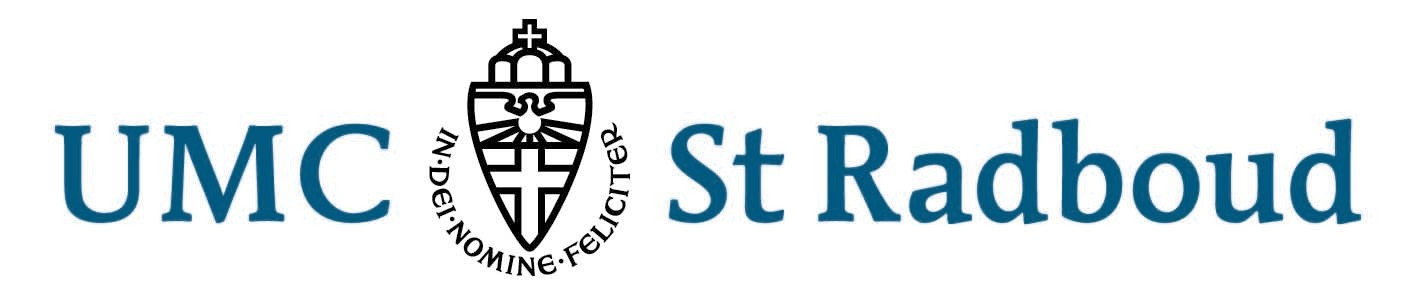


**Questionnaire patients:**

**Decision making in the advanced stage of Parkinson’s disease**

**U can also fill in the questionnaire online through the link below:**

**xxx**

**Your inlogcode is: xx**

This questionnaire has been developed by the Radboudumc as part of the project: shared decision making in advanced Parkinson’s disease. The questionnaire is based on earlier focus groups, interviews and literature study.

**Introduction to the questionnaire**

The completion takes approximately 30-45 minutes. You do not have to complete the questionnaire at once, to avoid that you become tired. The intention is that you always choose 1 answer unless it is indicated that you can choose multiple answers.

This questionnaire consists of three parts:

Part 1: here we ask questions about yourself.

Part 2: here we ask about your experiences at the moment that you chose one of the treatments.

Part 3: here we ask for your experiences with an active role in choosing the treatment.

**Terminology**

**Advanced treatment:** there are three advanced treatments: apomorphine pump, deep brain stimulation (DBS), and levodopa via an intestinal gel (Duodopa®). These are treatments that are started after (and in addition to) treatment with pills and all three treatments can improve Parkinson’s symptoms when the pills no longer work adequately.

The **apomorphine pump** is a pump that is connected to a tube that is inserted just below the skin with a small needle. Apomorphine is a drug that mimics the action of dopamine. The apomorphine pump delivers apomorphine non-stop into your body. This is different from the apomorphine injections where you inject the apomorphine with short duration.

**Deep brain stimulation (DBS):** You will sometimes only see the abbreviation in the text. DBS stands for deep brain stimulation. This is a brain operation where electrodes are placed into the brain that are stimulated by a stimulator (a kind of pacemaker that works continuously).

**Levodopa intestinal gel (Duodopa®):** The active substance is levodopa. Now it is introduced as a liquid substance through a tube directly through the abdominal wall into the stomach. That tube is called a PEG tube. And so the levodopa is continuously introduced through a tube into the stomach.

A **decision aid** is meant to help you choose one of these three advanced treatments. In a decision aid, information is given about all treatment options in a clear way. In addition, a step-by-step plan helps you as a patient find which treatment best suits your personal preferences. In other words, the decision aid helps you to make your preference clear so that you can discuss this with your neurologist.

**Part 1. General information**

1. What is your gender?

- Male
- Female

1. What is your age?

………………. years old

1. What is your living situation?

- Single / widow / widower
- Partner, living together / married
- Partner, not living together

1. What is your highest level of education? ( It is a standard classification of the educational system in the Netherlands)
   - Basisschool ( primary school)
   - Lbo/mavo/vmbo/mbo-1 (secondary education, pre-vocational education)
   - Havo/vwo/mbo2-4/beroepsopleiding/vakopleiding ( Secondary education, vocational education)
   - Hoger beroepsonderwijs (hbo)/ Wetenschappelijk onderwijs (higher education/ academic education)
2. Are you currently working (including volunteering)?

- Yes, fulltime
- Yes, parttime
- No

1. How long do you have Parkinson’s disease?

- Less than 5 years
- 5-10 years
- More than 10 years

1. Which statement best describes your current disease stage?

- There are no signs of Parkinson's disease
- Signs of Parkinson's disease can be seen on one side of my body
- Signs of Parkinson's disease can be seen on both sides of my body. I have no problems with my balance
- There are moderate signs of Parkinson's disease on both sides of my body. I have some problems with my balance. I can function physically independent
- I am severely restricted. I am able to walk or to stand without help.
- I am tied to a wheelchair or lie in bed unless I get help.

1. Where are you being checked for your Parkinson’s disease? We will only use this to determine the type of hospital. We will therefore only use this anonymously in our analysis. If you are being treated in several hospitals for Parkinson’s disease, you can state more hospitals.

Name of the hospital :. . . . . . . . . . . . . . . . . . . . . . . . . .

Place:. . . . . . . . . . . . . . . . . . . . . . . . . . . . . . . . . . . . . . . . . .

**Part 2: Choosing your treatment**

Three treatments are possible for patients in the advanced stage of Parkinson's disease:

1. Apomorphine pump: this is a medicine that is inserted through a tube just below the skin and where the medicine is given continuously via a pump.
2. Deep brain stimulation (DBS): this is a brain operation in which electrodes are inserted into the brain. The electrodes are stimulated through a stimulator that is placed under the skin near the chest.

3) Levodopa via intestinal gel (Duodopa®), this is levodopa that is introduced directly into the stomach through a tube through the abdominal wall. The drug is delivered continuously via a pump.

1. Which of the above treatments do you have you or have you had? (Multiple answers are correct)

- Apomorphine pump, started. . . . . . . . . . . . . . . (year)
- DBS, started. . . . . . . . . . . . . . . (year)
- Levodopa via intestinal gel (Duodopa®), started. . . . . . . . . . . . . . . (year)

1. Do you still have that/those treatment (s)?

- Yes
- No, stopped in. . . . . . . . (year)

1. You have received one of the treatments specified in the box above. Which of the treatment options were you aware of before you made your choice? (so you can tick multiple answers)

- Apomorphine pump
- DBS
- Levodopa via PEG tube (Duodopa®)

1. Imagine you have to make the decision again for an advanced treatment. What role would you like when making the decision? You will see 5 options below. Choose the option that suits you the most.

| 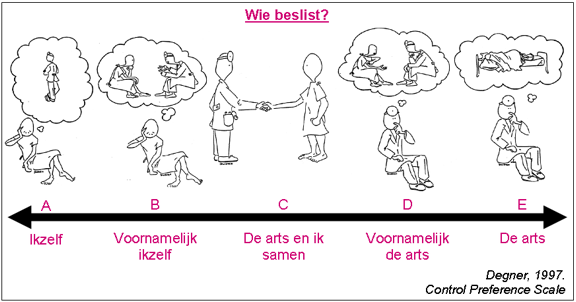  Me | 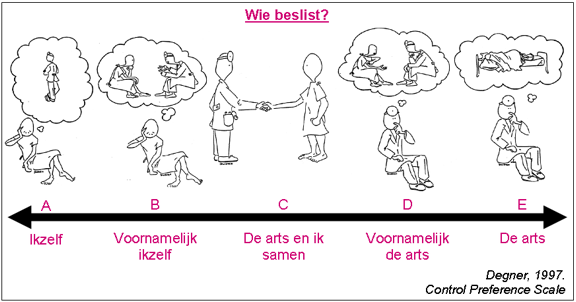  Mainly me | 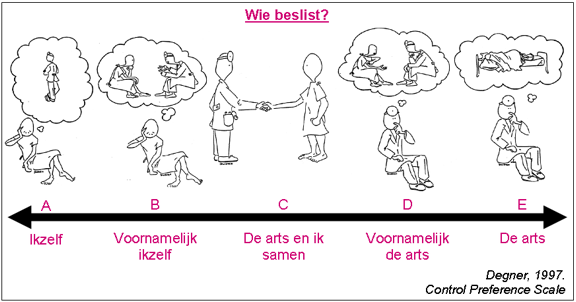  Me and the neurologist together | 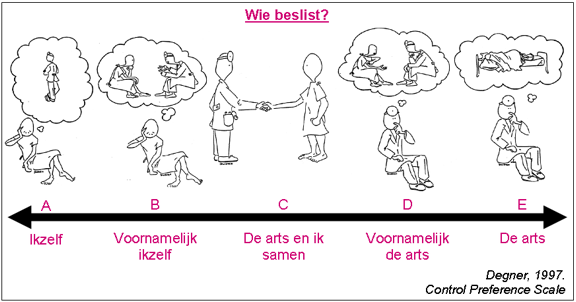  Mainly the neurologist | 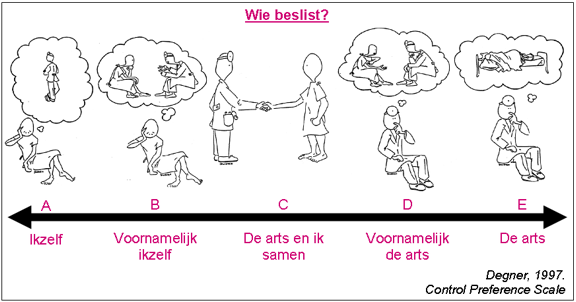  The neurologist |
| --- | --- | --- | --- | --- |
| ❑ | ❑ | ❑ | ❑ | ❑ |
| I prefer to make the final treatment decision | I prefer to make the final treatment decision, after seriously considering my doctor’s opinion | I prefer that my doctor and I make the final treatment decision together | I prefer the doctor to make the final treatment decision, after seriously considering my opinion | I prefer the doctor to make the final treatment decision |

1. . Now the question is what role you had when you made the choice. Choose the option that fits best.

| Referral | 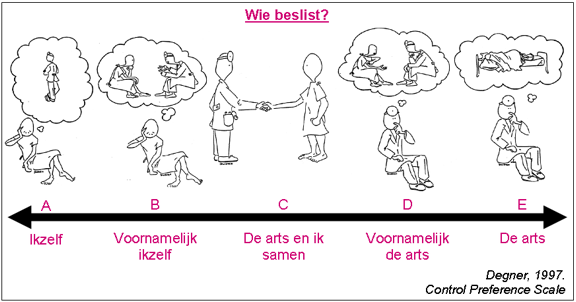  Me | 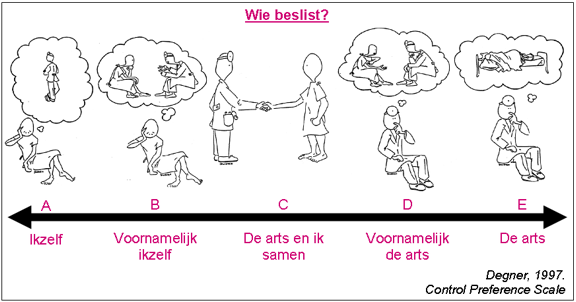  Mainly me | 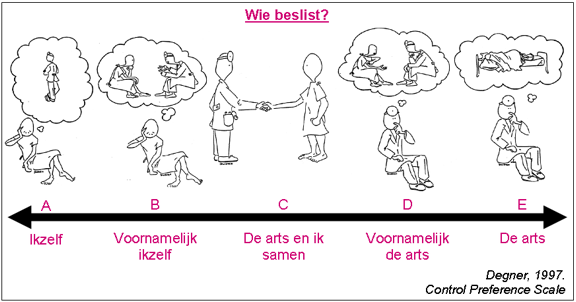  Me and the neurologist together | 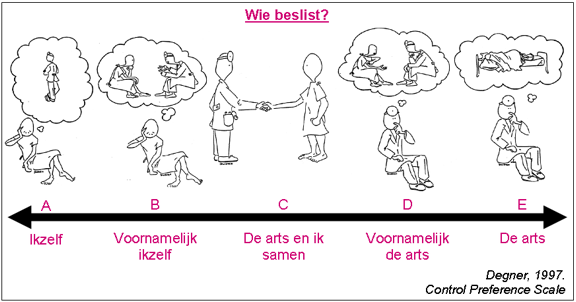  Mainly the neurologist | 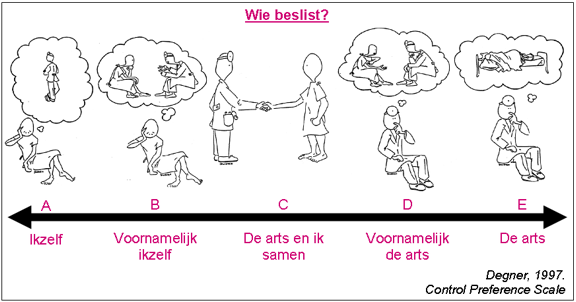  The neurologist |
| --- | --- | --- | --- | --- | --- |
| ❑ | ❑ | ❑ | ❑ | ❑ | ❑ |
| my neurologist and I haven't made a decision. I was referred to another specialist who made the choice (with me) | I made the final treatment decision | I made the final treatment decision, after seriously considering my doctor’s opinion | My doctor and I made the final treatment decision together | The doctor made the final treatment decision, after seriously considering my opinion | The doctor made the final treatment decision |

14. The following questions consist of two parts. What information did you receive from the neurologist or Parkinson's nurse specialist and how important is the information below in your opinion to be able to make a choice? So for each information item you answer whether you received that information before you made a choice and you answer how important you think that information is to make a choice.

|  | **Have you received information on this topic from the PD nurse specialist or neurologist?** | | | | | | | | | | **How important is this information to you?** | | | | | | |  | |
| --- | --- | --- | --- | --- | --- | --- | --- | --- | --- | --- | --- | --- | --- | --- | --- | --- | --- | --- | --- |
| **Information topic** | **I got sufficient information** | | **I got insufficient information** | | | | | **I got no information** | | **I don’t know** | **Very important** | **Important** | **Neutral** | **Not really important** | | **Not important** | | | |
| 1. Reasons why you **are** eligible for a treatment | ❑ | | ❑ | | | | | ❑ | | ❑ | ❑ | ❑ | ❑ | ❑ | | ❑ | | |  |
| 1. Reasons why you **are not** eligible for a treatment | ❑ | | ❑ | | | | | ❑ | | ❑ | ❑ | ❑ | ❑ | ❑ | | ❑ | | |  |
| 1. Expected psychological effects of a treatment | ❑ | | ❑ | | | | | ❑ | | ❑ | ❑ | ❑ | ❑ | ❑ | | ❑ | | |  |
| 1. Expected physical effects of a treatment | ❑ | | ❑ | | | | | ❑ | | ❑ | ❑ | ❑ | ❑ | ❑ | | ❑ | | |  |
| 1. Effects of a treatment on your daily activities | ❑ | | ❑ | | | | | ❑ | | ❑ | ❑ | ❑ | ❑ | ❑ | | ❑ | | |  |
| 1. Effects of a treatment on your quality of life | ❑ | | ❑ | | | | | ❑ | | ❑ | ❑ | ❑ | ❑ | ❑ | | ❑ | | |  |
|  | **Have you received information on this topic from the PD nurse specialist or neurologist?** | | | | | | | | | | **How important is this information to you?** | | | | | | | |  |
|  | **I got sufficient information** | **I got insufficient information** | | | | | **I got no information** | | | **I don’t know** | **Very important** | **important** | **Neutral** | **Not really important** | **Not important** | | | |  |
| 1. Possible complications/side –effects of a treatment | ❑ | | | | ❑ | | | | ❑ | ❑ | ❑ | ❑ | ❑ | ❑ | ❑ | | | |  |
| 1. Daily care needed for a treatment | ❑ | | | | ❑ | | | | ❑ | ❑ | ❑ | ❑ | ❑ | ❑ | ❑ | | | |  |
| 1. New tasks for a partner with a new treatment | ❑ | | | | ❑ | | | | ❑ | ❑ | ❑ | ❑ | ❑ | ❑ | ❑ | | | |  |
| 1. Screening procedure for a treatment | ❑ | | | | ❑ | | | | ❑ | ❑ | ❑ | ❑ | ❑ | ❑ | ❑ | | | |  |
| 1. Operation procedure | ❑ | | | | ❑ | | | | ❑ | ❑ | ❑ | ❑ | ❑ | ❑ | ❑ | | | |  |
| 1. setting-up/ adjusting a treatment | ❑ | | | | ❑ | | | | ❑ | ❑ | ❑ | ❑ | ❑ | ❑ | ❑ | | | |  |
| 1. Follow-up/ check-ups for a treatment | ❑ | | | | ❑ | | | | ❑ | ❑ | ❑ | ❑ | ❑ | ❑ | ❑ | | | |  |
| 1. Advantages/ disadvantages of a treatment | ❑ | | | | ❑ | | | | ❑ | ❑ | ❑ | ❑ | ❑ | ❑ | ❑ | | | |  |
|  | **Have you received information on this topic from the PD nurse specialist or neurologist?** | | | | | | | | | | **How important is this information to you?** | | | | | | | |  |
|  | **I got sufficient information** | | | **I got insufficient information** | | | | **I got no information** | | **I don’t know** | **Very important** | **important** | **Neutral** | **Not really important** | **Not important** | | | |  |
| 1. Consequences of a treatment for the parkinson pills | ❑ | | | | | ❑ | | ❑ | | ❑ | ❑ | ❑ | ❑ | ❑ | ❑ | |  | | |
| 1. Long term effects of a treatment | ❑ | | | | | ❑ | | ❑ | | ❑ | ❑ | ❑ | ❑ | ❑ | ❑ | |  | | |
| 1. Waiting list for a DBS operation | ❑ | | | | | ❑ | | ❑ | | ❑ | ❑ | ❑ | ❑ | ❑ | ❑ | |  | | |
| 1. Effects of reducing/discontinuing parkinson pills | ❑ | | | | | ❑ | | ❑ | | ❑ | ❑ | ❑ | ❑ | ❑ | ❑ | |  | | |
| 1. Consequences if a new treatment is stopped again | ❑ | | | | | ❑ | | ❑ | | ❑ | ❑ | ❑ | ❑ | ❑ | ❑ | |  | | |
| 1. Information on alternative treatments that are possible | ❑ | | | | | ❑ | | ❑ | | ❑ | ❑ | ❑ | ❑ | ❑ | ❑ | |  | | |
| 1. The reason for the neurologist's preference or choice | ❑ | | | | | ❑ | | ❑ | | ❑ | ❑ | ❑ | ❑ | ❑ | ❑ | |  | | |
| 1. Consequences of a treatment for partner | ❑ | | | | | ❑ | | ❑ | | ❑ | ❑ | ❑ | ❑ | ❑ | ❑ | |  | | |

In retrospect, you may have missed information (or guidance) that would have been important in making the decision, now that you have experience with the treatment.

1. Did you feel that you were fully informed at the time that you made a choice?

❑ Yes

❑ No, I missed. . . . . . . . . . . . . . . . . . . . . . . . . . . . . . . . . . . . . . . . . . . . . . . . . . . . . . . . . . . . . . . . . . . . . . . . . . . . . . . . . . . . . . . . . . . . . . . . . . . . . . . . . . . . . . . . . . . . . . . . . . . . . . . . . . . . . . . . . . . . . . . . .

1. Now that you have experience with one of the treatments, do you now think that you were fully informed at the time?

❑ Yes

❑ No, I missed. . . . . . . . . . . . . . . . . . . . . . . . . . . . . . . . . . . . . . . . . . . . . . . . . . . . . . . . . . . . . . . . . . . . . . . . . . . . . . . . . . . . . . . . . . . . . . . . . . . . . . . . . . . . . . . . . . . . . . . . . . . . . . . . . . . . . . . . . . . . . . . . .

**Part 3: Deciding together with your health care provider**

We think it is important that you as a patient can make the decision together with the neurologist if you want. With this research we want to develop a method to involve patients more actively in choosing a treatment. We would like to know if you also want to be actively involved and how that could be done. With making decisions together we mean:

1) That you as a patient and the neurologist are involved in choosing

2) That you share information together

3) That you take steps together to form a preference for a treatment

4) That you come to a decision together

The following are factors that make it easier for you to be actively involved as a patient.

Facilitating factors means: factors that make it easier. What is very important is that these factors are not facts, but factors mentioned by other patients in the discussion groups and from other studies. It may therefore also be that you do not agree with a factor.

**We would like to know which factors below helped you to participate actively in decision-making. By that we mean that facilitating factors are supporting you to join actively in the decision-making.**

| **facilitating factors patient** | **Large facilitating factor** | **Moderate facilitating factor** | **Small facilitating factor** | **No facilitating factor** | **No opinion** |
| --- | --- | --- | --- | --- | --- |
| 1. I could choose independently | ❑ | ❑ | ❑ | ❑ | ❑ |
| 1. I asked to be actively involved | ❑ | ❑ | ❑ | ❑ | ❑ |
| 1. The information was adapted to my personal needs | ❑ | ❑ | ❑ | ❑ | ❑ |
| 1. Because I had Parkinson's disease for a long time, I was already used to actively taking decisions | ❑ | ❑ | ❑ | ❑ | ❑ |

| **Facilitating factors professional** | **Large facilitating factor** | **Moderate facilitating factor** | **Small facilitating factor** | **No facilitating factor** | **No opinion** |
| --- | --- | --- | --- | --- | --- |
| 1. The neurologist or Parkinson nurse specialist actively encouraged / guided me to participate | ❑ | ❑ | ❑ | ❑ | ❑ |
| 1. The neurologist had the experience and the knowledge that guided my choice | ❑ | ❑ | ❑ | ❑ | ❑ |
| 1. There was a good relationship of trust between me and my neurologist and Parkinson nurse specialist | ❑ | ❑ | ❑ | ❑ | ❑ |
| 1. The neurologist or Parkinson nurse specialist and I needed each other's knowledge and experience and were therefore all actively involved | ❑ | ❑ | ❑ | ❑ | ❑ |
| 1. There was an open way of discussion between me and the neurologist or Parkinson nurse specialist | ❑ | ❑ | ❑ | ❑ | ❑ |
| 1. The neurologist took the time to discuss the choice with me | ❑ | ❑ | ❑ | ❑ | ❑ |
| 1. The neurologist involved my partner in the decision-making | ❑ | ❑ | ❑ | ❑ | ❑ |

| **Facilitating factors context / organization** | **Large facilitating factor** | **Moderate facilitating factor** | **Small facilitating factor** | **No facilitating factor** | **No opinion** |
| --- | --- | --- | --- | --- | --- |
| 1. There was neutral information on all treatments | ❑ | ❑ | ❑ | ❑ | ❑ |
| 1. The information was easy to find | ❑ | ❑ | ❑ | ❑ | ❑ |
| 1. There was written information | ❑ | ❑ | ❑ | ❑ | ❑ |

Below are factors that can make it harder for you to be actively involved as a patient. Limiting factors means: factors that make it harder to be actively involved. What is very important is that these factors are not facts, but factors mentioned by other patients in the discussion groups and from other studies, so you may also disagree with a factor.

We would like to know which factors below prevented you from actively taking part in decision-making. By that we mean which factors made it difficult to decide for yourself.

| **Limiting factors patiënt** | **Large limiting factor** | **Moderate limiting**  **factor** | **Small limiting factor** | **No limiting factor** | **No opinion** |
| --- | --- | --- | --- | --- | --- |
| 1. The information was not adjusted to me personally | ❑ | ❑ | ❑ | ❑ | ❑ |
| 1. There was too much information, which was confusing | ❑ | ❑ | ❑ | ❑ | ❑ |
| 1. I found the information I received difficult to use for my own situation because I did not have sufficient medical knowledge | ❑ | ❑ | ❑ | ❑ | ❑ |
| 1. I felt that there was no choice | ❑ | ❑ | ❑ | ❑ | ❑ |
| 1. I no longer had time to make a choice myself (because the symptoms were so bad that treatment had to be started quickly) | ❑ | ❑ | ❑ | ❑ | ❑ |
| 1. I was not ready for the choice yet | ❑ | ❑ | ❑ | ❑ | ❑ |
| 1. I thought it was too much responsibility to choose myself | ❑ | ❑ | ❑ | ❑ | ❑ |
| 1. I thought the neurologist had to choose, because that is the expert |  | ❑ | ❑ | ❑ | ❑ |
| 1. Me and my partner / caregiver had a different preference | ❑ | ❑ | ❑ | ❑ | ❑ |
| 1. I could not choose myself because I could not objectively judge my situation | ❑ | ❑ | ❑ | ❑ | ❑ |

| **Limiting factors professional** | **Large**  **limiting**  **factor** | **Moderate limiting factor** | **Small limiting factor** | **No**  **limiting factor** | **No opinion** |
| --- | --- | --- | --- | --- | --- |
| 1. The neurologist or Parkinson nurse specialist influenced me by the sort of information he / she provided | ❑ | ❑ | ❑ | ❑ | ❑ |
| 1. The neurologist or Parkinson nurse specialist did not have sufficient knowledge about the treatments | ❑ | ❑ | ❑ | ❑ | ❑ |
| 1. The neurologist had his own preference and did not consider the other options | ❑ | ❑ | ❑ | ❑ | ❑ |
| 1. Not all treatments were available in my own hospital, so the neurologist did not offer all treatments | ❑ | ❑ | ❑ | ❑ | ❑ |
| 1. The neurologist or Parkinson nurse specialist did not offer you to be actively involved | ❑ | ❑ | ❑ | ❑ | ❑ |
| 1. The different involved neurologists and Parkinson nurse specialists gave different advice | ❑ | ❑ | ❑ | ❑ | ❑ |
| 1. There was no constant neurologist or Parkinson nurse specialist | ❑ | ❑ | ❑ | ❑ | ❑ |

| **Limiting factors context / organization** | **Large limiting factor** | **Moderate limiting factor** | **Small limiting factor** | **No limiting factor** | **No opinion** |
| --- | --- | --- | --- | --- | --- |
| 1. There was not enough time at a consultation appointment to be able to discuss it properly | ❑ | ❑ | ❑ | ❑ | ❑ |
| 1. There was limited research available to compare treatments with each other | ❑ | ❑ | ❑ | ❑ | ❑ |

1. Do you have any additional factors that helped you to be able to make an active choice yourself or that made it difficult?

. . . . . . . . . . . . . . . . . . . . . . . . . . . . . . . . . . . . . . . . . . . . . . . . . . . . . . . . . . . . . . . . . . . . . . . . . . . . . . . . . . . . . . . . . . . . . . . . . . . . . . . . . . . . . . . . . . . . . . . . . . . . . . . . . . . . . . . . . . . . . . . . . . . . . . . . . . . .

. . . . . . . . . . . . . . . . . . . . . . . . . . . . . . . . . . . . . . . . . . . . . . . . . . . . . . . . . . . . . . . . . . . . . . . . . . . . . .

1. How did you fill in the questionnaire?

❑ I have completed the questionnaire alone

❑ I have completed the questionnaire together with my partner

❑ I have completed the questionnaire together with someone else namely:. . . . . . . . . .

. . . . . . . . . . . . . . . . . . . . . . . . . . . . . . . . . . . . . . . . . . . . . . . . . . . . . . . . . . . . . . . . . . . . . . . . . . .

(please state what role that person has, for example brother / sister, child)

Do you have any questions or comments based on this questionnaire about shared decision-making in treatments in the advanced stage of Parkinson's disease?

. . . . . . . . . . . . . . . . . . . . . . . . . . . . . . . . . . . . . . . . . . . . . . . . . . . . . . . . . . . . . . . . . . . . . . . . . . . . . . . . . . . . . . . . . . . . . . . . . . . . . . . . . . . . . . . . . . . . . . . . . . . . . . . . . . . . . . . . . . . . . . . . . . . . . . . . . . . . . . . . . . . . . . . . . . . . . . . . . . . . . . . . . . . . . . . . . . . . . . . . . . . . . . . . . . . . . . . . . . . . . . . . . . . . . . . . . .

These were the questions. Thank you for participating in this questionnaire! On the next page, please complete the consent form before you return this questionnaire.

On the basis of this questionnaire we evaluate how we can develop a decision aid for decision making in the advanced stage of Parkinson's disease. We will then test the decision aid for parkinson patients who are facing the decision.

Thank you again for your time and effort!

Sincerely,

On behalf of the research team,

Frouke Nijhuis

Physician/researcher
